# Some Details as to the Care of Dental Instruments

**Published:** 1894-12

**Authors:** William H. Potter

**Affiliations:** Boston


### SOME DETAILS AS TO THE CARE OF DENTAL INSTRUMENTS.[1]

BY WILLIAM H. POTTER, D.M.D., BOSTON.

It has always been my conviction that dental instruments should be as carefully cleansed and sterilized as are the instruments of the general surgeon. When but a student in dentistry, the prevalent disregard paid to the cleansing of instruments was a matter of surprise. I realized that dentists had dealings with infectious diseases of grave import, and yet I saw little concern among operators to make their instruments safe, as they were passed from mouth to mouth. And thus I was led to give the matter of the sterilization of dental instruments special study.

And here let me say, that I do not now propose to argue as to the desirability or necessity of sterilized instruments. I take that for granted, and believe that any one who will give the subject intelligent consideration will reach the same conclusion.

[1] Read before the Harvard Odontological Society, May 26, 1894.

During my first years of practice, I cared for my instruments in a manner resembling that which I saw in use at the hospitals of this city. There carbolic acid was the main reliance, in strength one to twenty. Following out this method, I used to wash my instruments as thoroughly as possible, and immerse them in a five per cent. carbolic acid solution. But rust would occur from prolonged immersion; and I considered prolonged immersion necessary for real germicidal effects. After using a five-per-cent. carbolic acid solution for several years, I changed to a ninety-five-per-cent. solution. This because of its more rapid germicidal effect, and because steel instruments could be long exposed to its influence without rusting.

By an arrangement of suitable trays I was enabled to handle my instruments without exposing my hands to the caustic effect of the acid. In connection with this process, I always associated to a considerable extent the use of boiling water, either with or without the addition of an alkali. The use of ninety-five-per-cent. carbolic acid, supplemented by the use of boiling water, was my reliance until Dr. Ernst, of the Harvard Medical School, read a paper before the Harvard Odontological Society, on the sterilization of dental instruments, and recommended the use of the Arnold sterilizer. This sterilizer is an exceedingly simple device for keeping a metal chamber at the temperature of 212° F. Articles put into this chamber are subjected to moist heat of such a temperature as to positively destroy any accompanying germs in a definite time. Whereas the germicidal power of carbolic acid or corrosive sublimate (however great it may be) is not as yet definitely determined; the germicidal power of moist heat at a given temperature has been accurately established by laboratory experiment. Following out the suggestion of Dr. Ernst, I provided myself with an Arnold sterilizer, and put it in operation. In the first use, I exposed my instruments to the direct effect of the steam as it was contained in the steam-chamber, and found that the process worked very nicely with such instruments as were protected by nickel plating; but that plain steel instruments were quickly rusted and ruined. I was forced on this account to give up the sterilizer as far as my steel instruments were concerned and return to the use of a ninety-five-per-cent. carbolic acid solution. Having been in the habit, before using the Arnold sterilizer, of boiling steel instruments in a solution of potassic hydrate and thereby sterilizing them without danger of rust, it occurred to me to combine the simple boiling process with the use of the sterilizer. And this was done in the following way: A zinc tray was made of suitable size to accommodate the instruments to be sterilized, and of proper shape to fit into the steam-chamber. My instruments were placed in this zinc tray and covered with a solution of potassic hydrate. Then the tank was placed in the sterilizer and subjected to the steam temperature for the proper time. The contents of the tank were thus sterilized without rusting steel instruments. Later carbonate of soda was substituted for potassic hydrate, because less expensive and equally effective. In this way I have used the Arnold steam sterilizer for over two years, and with very great satisfaction.

It is, however, difficult to satisfactorily treat certain of our instruments, as, for example, syringes. A syringe having the regulation packing of leather soaked in grease, evidently cannot be subjected to heat of any kind without ruining the packing, and necessitating an entirely new one. And yet how else can such syringes be kept really clean? The greased packing offers so many difficulties in the way of cleanliness that I have abandoned it altogether, except for the hypodermic syringe, and of that I will speak later. For my ordinary piston syringe, such as we use for flushing cavities and the common routine work, I make a packing from the material which composes felt wheels. This packing when touched with a little soap is an excellent substitute for a greased packing, and has this advantage, that it may be put in the sterilizer many times without impairing its usefulness. It cannot be immersed in the soda solution of which I have spoken, but can be exposed openly in the steam-chamber. I do not claim that a syringe with this packing is equal, as a mechanical instrument, to one with the regular greased packing. This more surely than anything else makes a water-tight joint. But I claim for the felt packing that it is cleanly and is sufficiently tight for all ordinary purposes. I might add here that it is my custom to put my syringe through the sterilizer after each use. Another syringe which I believe in is made by tying a rubber bulb, such as comes with a medicine dropper, about a Farrar syringe point. Such a syringe is very useful in the delicate work of treating roots and abscesses, and can be thoroughly cleansed by being taken apart and subjected either to boiling water or the sterilizer. My hypodermic syringe I fit out with a wash leather packing soaked in hot mutton tallow. After use I remove the packing and boil all parts of the syringe in a soda solution, and pack again for further use. My rubber dam straps and holders are sterilized after each use. The only instruments which I cannot readily sterilize are the automatic plugger-engine hand-piece and the mouth-mirror. Mechanical cleanliness must in the main serve for these instruments.

After instruments have been cleansed and sterilized, it is important that they be properly arranged for work. How can we avoid contaminating our clean instruments with unclean hands while in the midst of an operation? Shall we stop and cleanse the hands before reaching to the case for an instrument? Certainly we should, unless we can approach each instrument individually, without touching those which surround it. If instruments are packed closely in drawers and cases, then either we must laboriously cleanse our hands before approaching our cases, or else operate in an uncleanly manner. My solution of this difficulty is as follows: I provide a table of convenient height and size, cover it with a towel, and lay out upon its surface my working force of instruments. All my small instruments are placed on metal racks, each instrument having room enough about it to be handled without contaminating another. Many of the instruments, such as pliers, scissors, and syringes, are laid out in an orderly way upon the towel. But each instrument is by itself and can be picked up from the table, even with hands right from a patient's mouth, without soiling a neighboring instrument. This arrangement has given me the greatest satisfaction, because it is at once cleanly and rapid. My instruments are all under my hand, and do not have to be sought for in numerous drawers and cases. My operating-bracket consists of a plate of brass having a raised rim. It can be kept cleaner than one made of wood. I do not believe in keeping instruments in an operating-bracket. They are subject to the *débris* of every operation.

In closing, I cannot emphasize too strongly the necessity of having a supply of running water very convenient to the operating chair. It should not be more than five feet away, and not in a closet.

If it is inconveniently placed it will be too seldom used.

The form of faucet which I employ seems to me especially adapted for our work. It is made by cutting off the tops of two high arching faucets, such as are commonly used in china closets, uniting the lower parts with a horizontal tube, and inserting in the middle of this one of the cut-off tops. This gives the hot and cold water mixed from a single orifice, and places the discharge high up in a position most convenient for the hands.

